# Bioactive Edible Coatings for Fresh-Cut Apples: A Study on Chitosan-Based Coatings Infused with Essential Oils

**DOI:** 10.3390/foods14132362

**Published:** 2025-07-03

**Authors:** Nuzra Ali, Eredina Dina, Ayten Aylin Tas

**Affiliations:** Department of Health Professions, Faculty of Health and Education, Manchester Metropolitan University, Manchester M15 6BX, UK; nuzra.ali@stu.mmu.ac.uk (N.A.); eredina.dina@stu.mmu.ac.uk (E.D.)

**Keywords:** fresh-cut apples, chitosan, essential oils, oregano, cinnamon leaf, shelf life

## Abstract

This study developed chitosan-based active edible coating formulations with antioxidant and antimicrobial properties exhibited by oregano and cinnamon leaf essential oils (EOs) to extend the shelf life of fresh-cut ‘Braeburn’ apples. The primary coating consisted of chitosan (1.5% *w*/*v*), ascorbic acid (2% *w*/*v*), and citric acid (2% *w*/*v*). Oregano (0.06 and 0.15% *v*/*v*) and cinnamon leaf (0.06 and 0.1% *v*/*v*) EOs were added to the primary coating. The coated apple slices were stored for 9 days at 4 ± 1 °C. Changes in weight loss, water activity, titratable acidity, total soluble solids content, polyphenol oxidase (PPO) activity, firmness, colour, visual appearance, surface morphology, and microbial activity were measured on days 2 and 9. The results revealed that the control samples deteriorated rapidly during storage. However, higher concentrations of EOs reduced moisture loss, water activity, and acid conversion but slightly impacted visual appearance. The coatings effectively inhibited the PPO activity through storage. The formulation with 0.1% cinnamon leaf EO may be considered a viable candidate for application as a coating material, followed by the formulation containing 0.06% oregano EO, maintaining the optimum quality parameters of fresh-cut apples. Chitosan-based coatings with added EOs can be a promising alternative for maintaining fresh-cut apple quality during storage.

## 1. Introduction

Apples (*Malus domestica*) are one of the most cultivated climacteric fruits globally, with a high content of antioxidants and other nutrients and an excellent source of dietary fibre [1,2,3]. Being climacteric, apples undergo continuous ripening after harvest. Moreover, the post-harvest technological, mechanical, and environmental stresses on fresh-cut (FC) apples, such as picking, cutting, and slicing, accelerate tissue senescence by exposing internal tissues to oxygen, promoting ethylene production, and leading to rapid browning [4]. One of the most significant problems with FC apples is enzymatic browning, primarily caused by the activity of polyphenol oxidase (PPO) and peroxidase (POD) enzymes on phenolic compounds exposed during cutting [5,6]. This browning not only affects the visual appeal but also reduces consumer acceptance [7,8,9]. FC apples lose moisture rapidly, leading to a decline in firmness and crispness. This is exacerbated by the disruption of cellular integrity during cutting, which accelerates water loss and softening [10]. Cutting increases the surface area and exposes internal tissues, making fresh-cut apples more vulnerable to microbial contamination. This can lead to spoilage and potential food safety concerns. Exposure to oxygen after cutting can lead to oxidative degradation of nutrients, particularly vitamin C and phenolic compounds, reducing the nutritional quality of the product [10,11,12,13]. Therefore, FC apples have a shorter shelf life compared with unprocessed raw whole apples [14,15,16,17]. Extending the shelf life of FC apples while simultaneously maintaining product quality has become a focal point [18].

Numerous applications like edible coatings (ECs), ultraviolet (UV) irradiation, ozonation, and modified atmospheric packaging (MAP) have been implemented in the past years to enhance the shelf life of FC apples [7,12,18,19]. Among these, edible coatings are considered one of the efficient techniques used to retain the quality of FC apples. ECs are prepared using edible biopolymers and are usually classified as polysaccharide-, lipid-, or protein-based, used either alone or in combination to enhance the positive properties of the final fresh produce [13,20,21]. The thin layer that ECs create generates a semipermeable barrier to gases, water vapour, and solute movement, which helps to reduce the exposure to oxygen, moisture loss, and degradation process during storage [13]. In addition, ECs work effectively as a carrier for active compounds such as antimicrobials, antioxidants, and anti-browning agents such as essential oils and nano-emulsions, interacting with the surface of the food [13,22,23]. Therefore, ECs acting as carriers of bioactive compounds can help maintain quality, delay ripening, and extend the shelf life of fresh produce [13].

Chitosan is a carbohydrate-based biopolymer derived from chitin and is well known for its film-forming ability and antifungal and antibacterial properties [24,25]. Chitosan-based edible coatings have great potential to preserve fruits and vegetables [26]. Chitosan mainly helps reduce microbial spoilage and control respiration rates, which also helps in retaining firmness [25]. Other properties that make chitosan a satisfactory coating include the selective permeability to gases, including O_2_ and CO_2_ exchange, while retaining the mechanical strength of the coating [18,27].

Citric acid (CA) and ascorbic acid (AA) are commonly used in chitosan-based ECs because they aid chitosan in becoming soluble in water by slightly reducing the pH [28]. Moreover, these acids prevent browning and can exhibit antimicrobial properties as well as mild antioxidant properties that help maintain texture and colour without affecting taste and flavour [29,30]. The combination of chitosan and ascorbic acid coatings during cold storage (5 °C) was found to suppress browning and preserve the quality of fresh-cut apples, likely by inhibiting PPO activity [26].

Essential oils (EOs) are natural, volatile, aromatic metabolites—mainly complexes of terpenes and terpenoids—obtained from plants and used widely in ECs. They are considered GRAS (Generally Recognised as Safe), exhibiting antimicrobial, anti-inflammatory, and antioxidant properties [31]. They can also replace chemical preservatives in food [32]. Incorporating EOs as natural antimicrobial and strong antioxidant agents in ECs has been shown to extend the storage life of perishable foods [33,34]. Despite their advantages, using EOs is challenging because of their high reactivity and intense aroma [35]. Therefore, developing an optimised low-dose combination of EOs is essential to maintain the product quality and shelf life, while minimising the undesirable flavour and sensory changes associated with high concentrations [36].

Oregano essential oil (OEO) is a concentrated extract derived from the leaves of the oregano plant (*Origanum vulgare*), which is rich in carvacrol and thymol as well as monoterpene hydrocarbons, c-terpinene, and p-cymene, with antifungal, antimicrobial, and antioxidant properties [37,38]. The antioxidant and antimicrobial activity of OEO is primarily attributed to carvacrol, which is the major component [39]. Cinnamon essential oil (CEO) is extracted from the stems/bark of the cinnamon tree (*Cinnamomum zeylanicum*). It is a widely used EO due to its strong antifungal, antibacterial, and antioxidant properties and distinct aroma [40].

Chitosan-based edible coatings have been widely studied for their ability to preserve the quality of FC apples, particularly when combined with antioxidant and antimicrobial agents such as ascorbic acid, calcium salts, and EOs. Several studies have demonstrated the effectiveness of chitosan formulations in reducing enzymatic browning, maintaining firmness, and enhancing microbial stability in FC apples [17,26,41,42,43]. While cinnamon and oregano EOs have also been investigated for their preservative properties in FC apple coatings, these have primarily been incorporated into alginate- [44,45,46] or starch-based matrices [47,48]. To our knowledge, using chitosan as a carrier for these EOs in FC apple applications remains mostly uninvestigated, apart from one study that employed chitosan and cinnamon EO for the preservation of Golden Delicious apple slices [43]. Although chitosan-cinnamon EO coatings have shown promise in whole apple preservation [49,50,51], their potential in minimally processed apples has not been fully explored.

Therefore, the present study aimed to develop and evaluate chitosan-based edible coating formulations enriched with oregano and cinnamon leaf essential oils as natural antioxidants and antimicrobial agents. The objective was to assess their effectiveness in extending the shelf life of FC apples while preserving physicochemical quality and ensuring microbial safety.

## 2. Materials and Methods

### 2.1. Materials

Braeburn apples (*Malus domestica*) (origin: South Africa; time from harvesting to packaging: 12 h; Class 1; size: 63–68 mm), which were free from infection and physical defects, were purchased from a local market (Manchester, UK). Chitosan was purchased from Bulksupplements.com (UK). Citric acid was procured from BuyWholefoodsOnline.co.uk, and ascorbic acid was procured from specialingredients.co.uk. Oregano (grown in France) EO and cinnamon leaf (grown in Sri Lanka) EO were purchased from Healthy Supplies (Lancing, UK). The samples were labelled as shown in Table 1.

### 2.2. Preparation of Coating Solution

EC solutions were prepared by mixing 1.5% chitosan (*w*/*v*), 2% ascorbic acid, and 2% (*w*/*v*) citric acid within distilled water (primary coating). The solutions were heated in a water bath (SousVide Supreme Demi, Broomfield, CO, USA) for approximately 30 min at a constant temperature of 80 °C while stirring continuously until the solutions became transparent. Subsequently, the solutions were left to cool down at room temperature for 1 h.

To ensure the mixing of the primary coating with the EOs, Tween 20 (0.2%, *v*/*v*) was added as an emulsifying agent for the water-based solution while constantly stirring for 15 min on a stirring plate (Fisherbrand, Isotemp, Chengdu, China). Afterwards, oregano and cinnamon leaf essential oils (OEO 0.06 and 0.15%; CLEO 0.06 and 0.1%) were added to the coating solutions and stirred for 15 min. The concentration of the applied EOs was an average of what has been reported in the literature [18,52].

### 2.3. Preparation of Fresh Apples

A total of 4 kg (36 apples) of uniformly sized apples (each apple weighed approximately 100 g) were rinsed with distilled water for 5 min and left on sterile perforated trays for 1 h to dry. Afterwards, whole apples (with skin) were cut longitudinally into equal halves from the centre with a knife. Then, the halves were cut into uniform slices with dimensions of approximately 54 mm (height), 66 mm (width), and 21 mm (thickness) using a mandoline slicer (Modele at Marque, Lille, France).

### 2.4. Application of Coating

The apple slices were dipped in the solutions (Table 1) for 30 s under aseptic conditions and placed on sterile perforated trays. The slices were dried for 1 h at room temperature (24 °C) and covered with low-density polyethene (LDPE) films before being transferred into a chilled area (4 °C ± 1 °C, 65% relative humidity, RH) and stored for 9 days. The physicochemical and microbiological properties of samples were analysed on days 2 and 9. Retailers recommend the shelf life of FC apples to be 1 to 2 days. The reason for choosing 2 and 9 days of storage was to investigate whether the coatings applied would extend the shelf life of the samples by a further 7 days.

### 2.5. Physicochemical Analysis

#### 2.5.1. Weight Loss

Each apple slice was weighed in triplicate (*n* = 3) using an analytical balance. Weight loss (WL) of the samples was calculated by Equation (1), and results were expressed as a percentage.

(1) $$\mathrm{WL}\;\left(\%\right)=\frac{\left(\mathrm{Wi}\;-\;\mathrm{Wf}\;\right)}{\mathrm{Wi}}\;\times \;100$$

where

WL—weight loss expressed as %,
W_i_ (g)—initial weight, day 0,
W_f_ (g)—final weight measured on day 2 and day 9.

#### 2.5.2. Water Activity (a_w_)

Water activity (a_w_) was determined using a meter (Rotronic HygroLab manual) and 6 to 7 g of finely cut apple slices. The measurements were performed at 24 °C (*n* = 3).

#### 2.5.3. Total Soluble Solids (TSS)

Samples (10 g) of apple slices were mashed using a garlic press to extract the juice (*n* = 3). The TSS content of the apple juice was measured using an Atago 7 Brix Refractometer (Tokyo, Japan).

#### 2.5.4. Titratable Acidity (TA)

The juice of 25 g of apples was extracted using a garlic press and then filtered using a muslin cloth. Then, 10 mL of the apple juice was added to 25 mL of distilled water. After adding 2–3 drops of 1% phenolphthalein indicator, the solution was titrated with 0.1 mol/L sodium hydroxide (0.1 N NaOH) standard solution until the colour changed to light pink, persisting for a few seconds. TA was calculated by Equation (2), and results expressed as percent malic acid:

(2) $$\mathrm{TA}\;\left(\%\right)=\frac{V_{0}\times \;N\;\times 0.067\times {\;V}_{1}}{m\;\times {\;V}_{2}\;}\times 100$$

where

V_0_—sample volume,
N—NaOH concentration,
0.067—conversion factor calculated using malic acid,
V_1_—volume of NaOH used,
m—weight,
V_2_—filtrate volume.

#### 2.5.5. Polyphenol Oxidase Activity (PPO)

Apple slices were cut into a fine dice and juiced using a manual juicer. Then, 10 mL of juice was collected for each sample, and 0.2 mL of juice (enzyme extract) was mixed with 3.2 mL phosphate buffer (0.1 M, pH 6.8). Afterwards, 0.6 mL of 3,4-dihydroxyl phenylalanine (DOPA) solution was pipetted into the enzyme/buffer solution. Absorbance readings were taken at 0, 1, and 2 min (*n* = 3) at 475 nm (Cole-Parmer SP-200 Visible Spectrophotometer, Cambridgeshire, UK). PPO activity was expressed as units per ml (U/mL).

#### 2.5.6. Texture Measurements

The firmness of the samples was measured at room temperature (20 ± 1 °C) using a TA texture analyser (TA-XT2, Stable Micro Systems, Godalming, UK) equipped with a 5 kg load cell and coupled with a 6 mm small cylindrical probe. Apple slices were placed on the texture analyser and compressed to a 2 mm penetration distance with an auto trigger force. Measurements were taken from different points on the slices (*n* = 3).

#### 2.5.7. Colour Measurements and Visual Appearance

The colour of the apple slices was measured (*n* = 3) using a Datacolor 800 Colourimeter (Datacolor UK Ltd., Newport, UK) to determine the L* value (lightness or brightness), a* value (redness or greenness), and b* value (yellowness or blueness). The colourimeter was calibrated using a white tile standard. Three apple slices were used per sample, and measurements were taken from two points on the same apple slice. Browning index (BI) was calculated by Equations (3) and (4):

(3) $$\mathrm{BI}=\frac{\left(X-0.31\right)\times 100}{0.172}$$

where

(4) $$X=\frac{\left(a^{*}+1.75L^{*}\right)\;}{\left(5.64L^{*}+a^{*}\right)-\left(3.02b^{*}\right)}$$

#### 2.5.8. Scanning Electron Microscopy (SEM)

Apple slices were subjected to a series of preparation steps before SEM analysis to ensure structural integrity under vacuum conditions. The samples were chopped, frozen, and then freeze-dried for 2 days at −109 °C using a LaboGene Scanvac Cool Safe freeze-drier (LaboGene ApS, Lynge, Denmark). No chemical fixation was performed; freeze-drying was used as a minimally destructive method to dehydrate the tissues while preserving their original shape and structure. This process increased cell porosity and minimised distortion or collapse during high-vacuum imaging. The freeze-dried samples were trimmed to appropriate sizes to fit onto the SEM specimen stubs (not exceeding the stub diameter) and were carefully positioned to ensure optimal beam interaction. No conductive coating was applied, as the samples were analysed in their non-conductive state.

SEM imaging was conducted using a Hitachi TM4000 Plus tabletop scanning electron microscope (Hitachi High-Tech Corporation, Tokyo, Japan). This microscope operates on the same fundamental principles as conventional SEMs, utilising electron beams and vacuum conditions to generate high-resolution images. The secondary electron detection mode (ETD) was used to capture the apple tissues’ detailed surface topography and morphological features.

The specimen height was adjusted using the instrument’s height gauge to minimise beam interference and optimise image quality. Imaging was performed under standard (M) vacuum conditions at an accelerating voltage of 15 kV. Stigmation and magnification (typically around a 300 µm field of view) were manually adjusted to enhance image clarity and resolution.

### 2.6. Microbiological Analysis

Plate Count Agar (PCA) and Malt Extract Agar (MEA) were used for total viable count (TVC) and yeast and mould count, respectively. Apples were weighed (10 ± 1 g) and placed aseptically in a sterile stomacher bag. To this, 90 mL of Buffered Peptone Water (BPW) was added to make a 1:10 dilution, and samples were homogenised using a stomacher. Serial dilutions were conducted in 9 mL BPW bottles. A volume of 0.1 mL was deposited on PCA and MEA plates and spread with sterile spreaders. Plates were inverted and incubated at respective times and temperatures (PCA 48 h at 37 °C; MEA 72 h at 30 °C).

### 2.7. Statistical Analysis

The data were analysed using SPSS version 29.0 (IBM, 2022). Independent *t*-tests were used to determine the effect of storage time (days 2 and 9) on the properties of the samples. A one-way ANOVA test was applied to compare variances between samples with different coatings at a confidence interval of 95%. A Bonferroni *post hoc* test was used for multiple comparisons of subsets with significant differences at the *p* < 0.05 level.

## 3. Results and Discussion

### 3.1. Physicochemical Analysis

#### 3.1.1. Weight Loss

**Figure 1:**
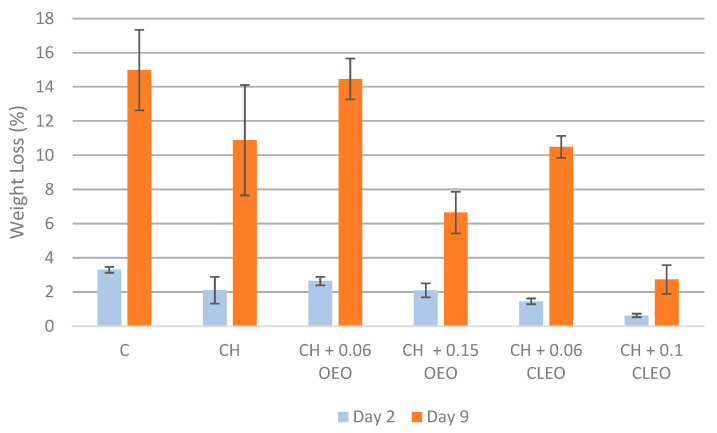
Weight loss (%) (*n* = 3): untreated apple slices (C), 1.5% chitosan (CH), 1.5% chitosan + 0.06% oregano essential oil (CH 0.06 OEO), 1.5% chitosan + 0.15% oregano essential oil (CH 0.15 OEO), 1.5% chitosan + 0.06% cinnamon leaf essential oil (CH 0.06 CLEO), and 1.5% chitosan + 0.1% cinnamon leaf essential oil (CH 0.1 CLEO) during storage at 4 °C. Error bars represent standard deviation (SD).

One of the most important indicators of the quality of FC fruits is the weight loss (WL). FC apples are susceptible to moisture loss during storage due to high transpiration and respiration rates, which immediately impacts their freshness [53]. Slicing apples exposes the skinless tissue to an environment with lower RH, causing substantial weight loss [17]. Control FC apple slices in the current study showed the highest WL, up to 15.0%, on day 9, as shown in Figure 1. This was because the uncoated FC apple slices lacked the physical barrier to protect them from environmental conditions. Conversely, chitosan-coated samples showed moderate WL. It was reasoned that the chitosan-based ECs, being hydrophilic, were less effective at retaining the moisture content of apples [54]. One study [26] reported that the WL of FC apples coated with 1% chitosan and AA (1 and 5%) was significantly lower than the control samples during 14 days of storage at 5 °C (*p* < 0.05), similar to our study. However, another study [55] reported no significant effect on the moisture loss in the apples coated with formulations containing AA or CA. The higher the concentration of EOs used, the less WL can be seen in the current study, maintaining the tissue firmness of the FC apples. Apple slices coated with 0.1% CLEO showed the least WL, 2.7%, followed by the 0.2% OEO coating with 6.6% WL during 9 days of storage.

Several studies reported the effectiveness of cinnamon EO incorporated into chitosan-based films or coatings in reducing WL in coated fruits and vegetables [43,56,57]. One relevant study specifically demonstrated that chitosan-based coatings with cinnamon EO significantly reduced WL in apple slices over 25 days of storage at 4 °C [43].

#### 3.1.2. Water Activity (a_w_)

Table 2 shows that a_w_ values ranged between 0.866 and 0.945, with CH 0.06 CLEO and CH 0.1 CLEO displaying the lowest a_w_ value on day 9. Incorporating lower concentrations of cinnamon EO (0.2%) in ECs proved to be an effective and potential solution due to its strong hydrophobic and antimicrobial properties, with a tendency to delay WL and result in a decrease in a_w_ values simultaneously [56]. This slowed down moisture migration, reduced the risk of microbial contamination, and extended the shelf life of FC apples [18]. In the current study, the control and samples CH, 1.5 CH 0.06 OEO, and 1.5 CH 0.15 OEO registered increased a_w_ values on day 9. An increase in a_w_ during cold storage (at 4 °C) indicates that there is more free (unbound) water available in apples, implying several issues like moisture migration, accelerated enzymatic browning, and an increased risk for microbial growth [58,59].

#### 3.1.3. Total Soluble Solids (TSS)

The TSS content of fruits is an essential factor reflecting their sweetness and quality, and the higher the TSS content, the sweeter the fruit is [53]. The increase in TSS content can be due to the re-conversion of starch into monosaccharides [60]. In the current study, all samples registered increased TSS concentrations over 9 days of storage. This was similar to what was obtained with Braeburn apples (7 to 14%) as reported by another study [61]. Table 2 shows that the control sample had the highest TSS value at 14.5% on day 2, with a minor increase to 14.8% on day 9. However, samples coated with 1.5% CH and OEO/CLEO showed the lowest TSS change, ranging from 12.0 to 12.6% on day 2. On day 9, all samples underwent a significant increase: the CH 0.15% OEO sample registered the lowest value (12.7%), and the highest TSS content was observed in the sample CH 0.06% CLEO (17.4%). One study [41] evaluated the effects of chitosan-based edible coatings combined with AA on FC apples. All coated samples, including those with chitosan-AA and chitosan-CaCl_2_, exhibited higher TSSs than the uncoated control. This suggests that while chitosan-AA coatings may moderately reduce TSS accumulation relative to other treatments, they may still lack sufficient capacity to effectively restrain metabolic activity and delay fruit decay in fresh-cut apples. Other quality parameters need to be considered for coating efficiency.

#### 3.1.4. Titratable Acidity (TA)

**Figure 2:**
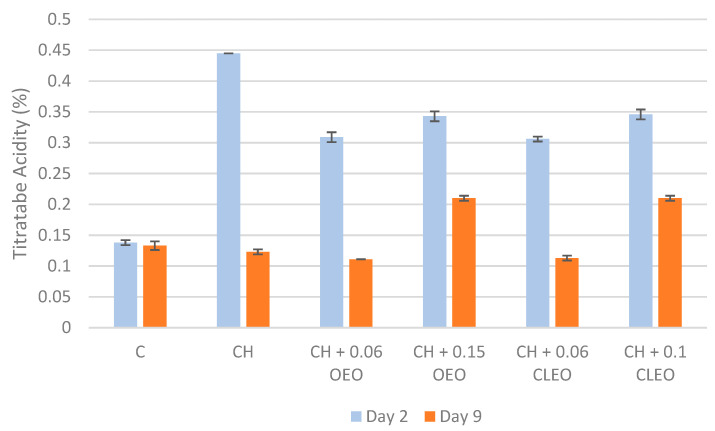
Titratable acidity values (%) (*n* = 3): untreated apple slices (C), 1.5% chitosan (CH), 1.5% chitosan + 0.06% oregano essential oil (CH 0.06 OEO), 1.5% chitosan + 0.15% oregano essential oil (CH 0.15 OEO), 1.5% chitosan + 0.06% cinnamon leaf essential oil (CH 0.06 CLEO), and 1.5% chitosan + 0.1% cinnamon leaf essential oil (CH 0.1 CLEO) during storage at 4 °C. Vertical error bars represent standard deviation (SD).

Changes in the TA of FC apples during storage mainly determine the sugar/acid ratio of apples, which is one of the primary indicators of their taste and ripeness [62]. During ripening, organic acids are consumed through respiration metabolism [63]. The respiration in FC apples also occurs due to minimal processes like peeling and cutting [43]. In the current study, uncoated samples had a slow conversion of acids into sugars (soluble solids), and the ripening of FC apples gradually decreased in 9 days of storage (Figure 2). On the contrary, one study reported a significant decrease in the TA of control FC apples from 0.4 to 0.1% during 15 days of storage at 4 °C [62]. In the current study, the TA content of coated samples showed a significant decrease from day 2 to 9. The findings were similar to another study [64], where the coating with chitosan and cinnamon bark EO resulted in lower TA in mangos compared with the uncoated sample during 18 days of storage at ambient temperature. In addition, in the current study, the TA content of samples with a higher EO concentration (CH 0.15 OEO and CH 0.1 CLEO) was higher than that of other samples on day 9. It can be suggested that these formulations could more efficiently control the respiratory processes of FC apples and thereby inhibit the consumption of acidic compounds within the apples [18,65]. Hence, 0.15% OEO and 0.1% CLEO concentrations can be considered relatively effective formulations to retain acidity in FC apples during extended storage.

#### 3.1.5. Polyphenol Oxidase (PPO) Activity

**Figure 3:**
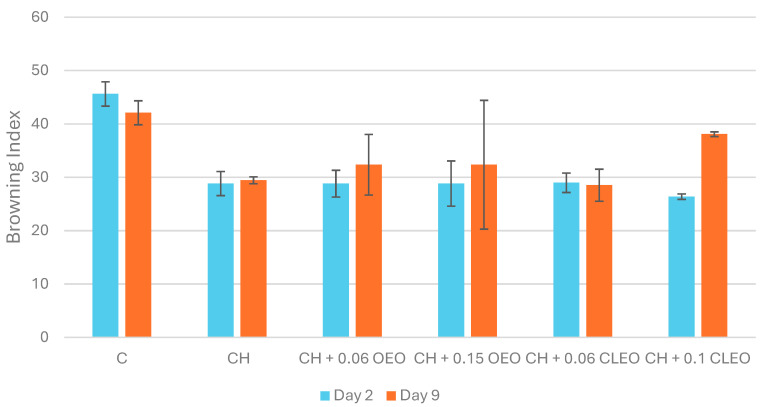
Browning index values (*n* = 3): untreated apple slices (C), 1.5% chitosan (CH), 1.5% chitosan + 0.06% oregano essential oil (CH 0.06 OEO), 1.5% chitosan + 0.15% oregano essential oil (CH 0.15 OEO), 1.5% chitosan + 0.06% cinnamon leaf essential oil (CH 0.06 CLEO), and 1.5% chitosan + 0.1% cinnamon leaf essential oil (CH 0.1 CLEO) during storage at 4 °C. Vertical error bars represent standard deviation (SD).

In fruits and vegetables, minimal processing such as peeling and chopping can easily lead to enzymatic browning [66]. Likewise, in the current study, high PPO activity was observed in apples within 5 min of slicing (30.1 ± 3.9 U/mL). However, the control and coated samples showed no PPO activity during storage (Table 2); therefore, browning index (BI) and colour analysis were used to elucidate the changes in the samples. The control sample showed the highest BI on day 2, and this value did not change significantly on day 9 (Figure 3), suggesting a higher PPO activity just after the cutting operation and a decline afterwards, evident from the coated samples registering comparatively lower BI values. Studies have also shown that PPO activity tends to peak initially, reach a maximum in uncoated samples, and then decline over 10 days of storage at 5 °C [17,67]. The PPO activity in untreated FC pear samples was documented to be 3 to 6 times higher, and coated samples had significantly lower values on days 5 and 10 of chilled storage (5 °C) [67]. Applying coatings of 1% chitosan with 5% AA and 2% chitosan with 2% AA proved effective in preventing enzymatic browning in fresh-cut apples for up to 14 days at 5 °C. [26]. Similar results were obtained in the present study, where coated samples stored at 4 °C did not exhibit any PPO activity. Therefore, it could be proposed that storing coated FC apples at lower temperatures (2–5 °C) can help slow down the rate of oxidation reactions by reducing PPO activity.

#### 3.1.6. Firmness

FC apples are prone to textural degradation due to the cellular changes propagated by enzymatic and non-enzymatic stresses caused by processes such as cutting and slicing [68]. In the current study, the control sample did not show a significant decrease in firmness during 9 days of storage (Table 2). The firm texture observed in uncoated apple slices during storage can be attributed to moisture loss driven by osmotic and evaporative processes [69,70]. Samples CH 0.06 OEO, CH 0.15 OEO, and CH 0.1 CLEO maintained firmness from day 2 to day 9; this agreed with other studies [43,71]. These studies featured oregano and cinnamon EOs in coatings to maintain the texture of FC apples by reducing WL and enzymatic and metabolic processes during cold storage, delaying the senescence of FC apples. One study [46] highlighted that edible coatings incorporating oregano and cinnamon EOs can effectively reduce water loss and enzymatic activity in FC Fuji apples, preserving tissue integrity and firmness during storage at 4 °C for 21 days. Similarly, another study [43] demonstrated that cinnamon EO-based coatings significantly maintained the firmness of minimally processed Golden Delicious apple slices stored at 4 °C for 25 days by minimising dehydration and delaying senescence-related softening. It has also been demonstrated that coatings (1% chitosan + 1% cinnamon EO) helped retain the firmness of guava fruit better than the control, but firmness still decreased progressively over time (at 5 °C for 21 days) [72]. Similar results were observed in the present study, where CH and CH 0.06 CLEO samples had the most significant decrease on day 9, which could result from moisture migration between the applied coating and fruit surface [73].

#### 3.1.7. Colour Measurements and Visual Appearance

Colour and visual appearance are the most important quality parameters for consumer acceptability [74]. In the current study, uncoated samples showed the highest BI (45.6) on day 2 (Figure 3). However, the chitosan-based coating (CH) registered the highest lightness (L*) (Table 3) and the lowest BI values, possibly acting as a bioactive semipermeable layer against oxygen and delaying browning [75]. Several studies achieved similar results [17,26,42]. All of these studies utilised AA (in addition to chitosan), which acted as an anti-browning agent, reducing o-quinones to o-diphenols and decelerating the formation of melanin, a browning compound [76].

**Figure 4:**
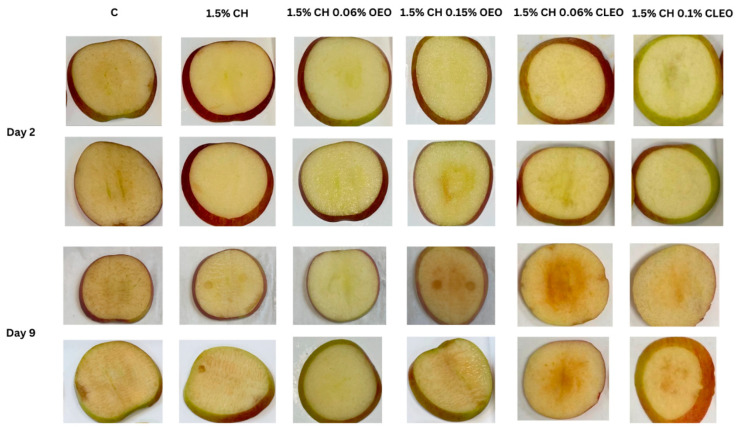
Visual appearance of FC apple slices: untreated apple slices (C), 1.5% chitosan (1.5% CH), 1.5% chitosan + 0.06% oregano essential oil (1.5% CH 0.06% OEO), 1.5% chitosan + 0.15% oregano essential oil (1.5% CH 0.15% OEO), 1.5% chitosan + 0.06% cinnamon leaf essential oil (1.5% CH 0.06% CLEO), and 1.5% chitosan + 0.1% cinnamon leaf essential oil (1.5% CH 0.1% CLEO) during storage at 4 °C.

Figure 3 illustrates the effect of different chitosan-EO coatings on the BI values. Notably, coatings with lower EO concentrations—specifically, samples CH 0.06 OEO and CH 0.06 CLEO—exhibited significantly lower BI values on both day 2 and day 9 than the control and higher EO concentrations. These treatments also maintained higher L values* (Table 3), indicating better preservation of lightness and reduced browning, as shown in Figure 4. This suggests that lower EO concentrations may offer an optimal balance between antimicrobial activity and barrier properties, effectively delaying enzymatic browning. These findings are consistent with another study [44], which documented that mild EO-based coatings helped maintain the visual quality of FC apples without inducing phytotoxic effects.

However, as the concentration of OEO increased, a sudden decline in the L* value was observed (Table 3). This may be caused by the phytotoxic effect of EOs leading to adverse cellular metabolic activity in the damaged cellular tissue (especially the middle, seed sections of slices, where high pressure was applied during cutting) as a natural defence mechanism against carvacrol, thymol, cinnamaldehyde, and eugenol found in EOs [77]. Similar results on phytotoxic effects on fresh produce were observed by other studies when oregano, cinnamon, and thyme EOs were used at higher concentrations [56,78]. The same immediate plant tissue damage was observed during the initial testing of formulations in the current study’s preliminary stages when 0.5 to 2% OEO and 0.5 to 2% CLEO were used. At higher concentrations, apple slices were observed to turn brown within an hour of the completion of the coating process.

#### 3.1.8. Scanning Electron Microscopy (SEM)

**Figure 5:**
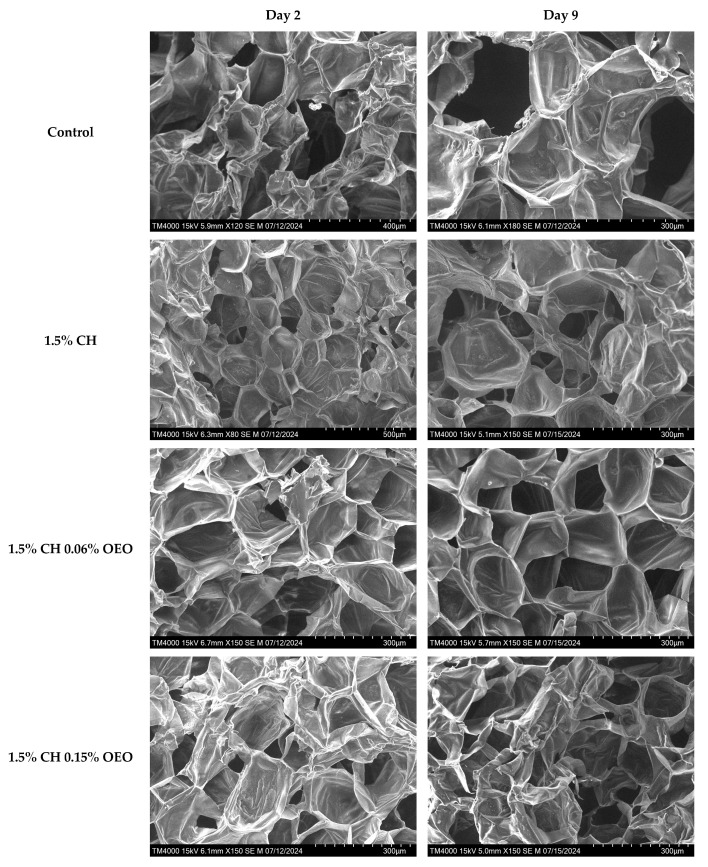

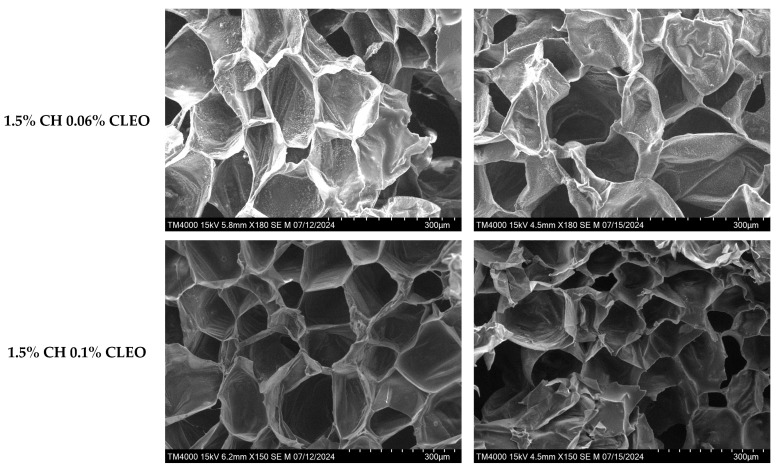
SEM analysis of FC apple samples: untreated apple slices (C), 1.5% chitosan (1.5% CH), 1.5% chitosan + 0.06% oregano essential oil (1.5% CH 0.06% OEO), 1.5% chitosan + 0.15% oregano essential oil (1.5% CH 0.15% OEO), 1.5% chitosan + 0.06% cinnamon leaf essential oil (1.5% CH 0.06% CLEO), and 1.5% chitosan + 0.1% cinnamon leaf essential oil (1.5% CH 0.1% CLEO) during storage at 4 °C.

SEM images help analyse material surface morphology and composition in depth. The images of samples at different magnifications in the current study exhibited a clear cellular structure (Figure 5). For instance, the control sample displayed many inter-tissue spaces and damaged cells, indicating high moisture loss and the lack of a barrier between the slices and the environment. On the contrary, all coated samples reflected smooth surfaces and intact cellular tissues, revealing efficient layer formation on the samples along with strong inter- and intra-molecular interactions between the constituents. The coating with 0.06% CLEO featured surface irregularities on day 2, and this could be explained by the lack of proper emulsification of the film solution and the oil droplets’ immiscibility, which can be seen on the sample surface [79,80,81,82]. Moreover, the multiple preparation steps before SEM analysis, including chopping, freezing, freeze-drying, and handling, might have damaged the delicate cellular tissues of the samples.

### 3.2. Microbiological Analysis

**Figure 6:**
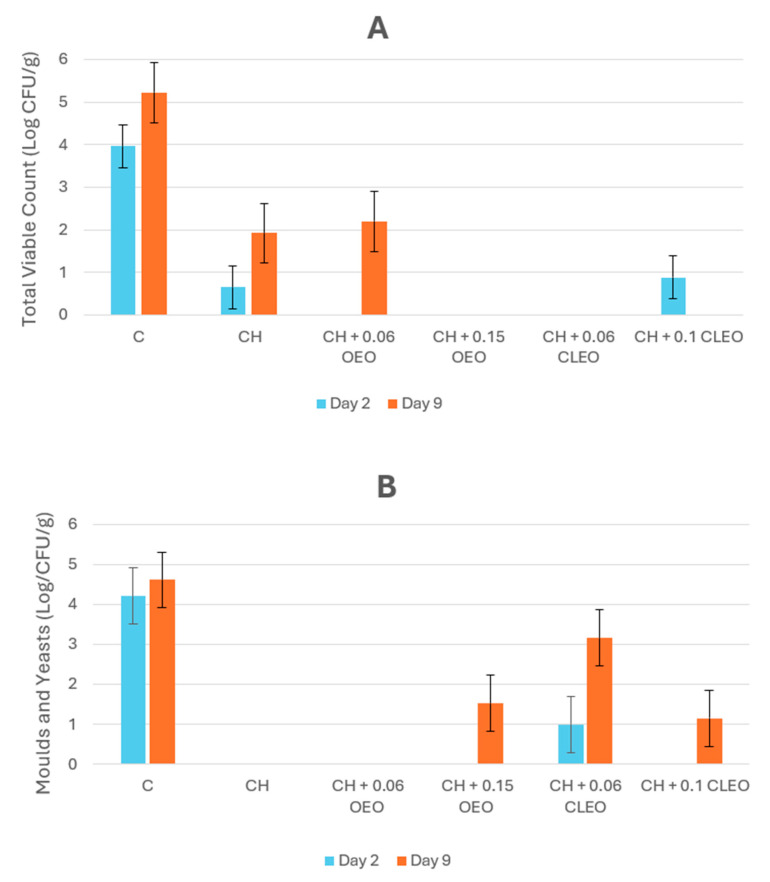
(**A**) Total viable count (TVC) (Log CFU/g) and (**B**) mould and yeast count (Log CFU/g) of untreated apple slices (C), 1.5% chitosan (CH), 1.5% chitosan + 0.06% oregano essential oil (CH 0.06 OEO), 1.5% chitosan + 0.15% oregano essential oil (CH 0.15 OEO), 1.5% chitosan + 0.06% cinnamon leaf essential oil (CH 0.06 CLEO), and 1.5% chitosan + 0.1% cinnamon leaf essential oil (CH 0.1 CLEO) during storage at 4 °C. Vertical error bars represent standard deviation (SD).

The microbial analysis showed a significant difference (*p* < 0.05) between the control and coated samples. Uncoated apple slices were highly prone to aerobic and fungal microorganisms even in cold storage, as presented in Figure 6. In contrast, coating efficiently halted the growth of aerobic bacteria, and fungal growth was prevented in samples CH and CH 0.06 OEO throughout storage. However, negligible mould growth on day 9 was observed in apple slices coated with 0.15 OEO and 0.06 and 0.1 CLEO; one of the reasons for this might be due to contamination during processing or improper handling of the samples. OEO sufficiently retarded fungal activity when compared with CLEO. This was attributed to the carvacrol content of the former and its strong lipophilic activity, which causes depletion of the cellular constituents of microorganisms, resulting in the inhibition of microbial growth [71]. The coating with a higher concentration of CLEO restricted fungal growth, consistent with other studies [49,56]. These studies documented that an increase in the concentration of cinnamon EO increased the antimicrobial activity of coatings because it interferes with the membrane permeability of microorganisms. Studies found that chitosan-based coatings worked effectively during the storage of FC apples [26] and candied kumquat [83] against various microorganisms, including mesophiles, psychrophiles, yeasts, moulds, and coliforms. Chitosan interacts with negatively charged microbial cell membranes, leading to increased permeability, leakage of intracellular contents, and ultimately, cell death [84].

## 4. Conclusions

This study successfully achieved its objective of developing and evaluating chitosan-based edible coatings enriched with oregano and cinnamon essential oils as natural antioxidants and antimicrobial agents for fresh-cut apples. Among the tested formulations, the combination of 1.5% chitosan with 0.1% cinnamon leaf essential oil demonstrated the most pronounced effectiveness in preserving the physicochemical and microbiological quality of Braeburn apple slices during refrigerated storage. The coating maintained the colour and firmness, reduced enzymatic browning and water activity, and inhibited microbial growth. A slightly lower but still notable efficacy was observed with the oregano EO formulation at 0.06%.

These results underscore the potential of such coatings as viable, biodegradable alternatives to synthetic preservatives in the fresh-cut fruit industry. Future research should focus on sensory evaluation, optimisation of coating application and slicing techniques, and integration with complementary preservation strategies such as modified atmosphere packaging to facilitate broader application and commercial scalability.

## Figures and Tables

**Table 1:** Sample descriptions and codes.

Coating/Essential Oil Used	Sample Description	Sample Code
No coating	Control	C
Chitosan only	1.5% Chitosan	CH
Chitosan and EOs	1.5% Chitosan + 0.06% Oregano EO	CH 0.06 OEO
	1.5% Chitosan + 0.15% Oregano EO	CH 0.15 OEO
	1.5% Chitosan + 0.06% Cinnamon leaf EO	CH 0.06 CLEO
	1.5% Chitosan + 0.1% Cinnamon leaf EO	CH 0.1 CLEO

**Table 2:** Water activity (a_w_), total soluble solids (TSS), polyphenol oxidase (PPO) activity, and firmness (N) of untreated FC apples (C), 1.5% chitosan (CH), 1.5% chitosan + 0.06% oregano essential oil (CH 0.06 OEO), 1.5% chitosan + 0.15% oregano essential oil (CH 0.15 OEO), 1.5% chitosan + 0.06% cinnamon leaf essential oil (CH 0.06 CLEO), 1.5% chitosan + 0.1% cinnamon leaf essential oil (CH 0.1 CLEO) during storage at 4 °C.

	C	CH	CH 0.06 OEO	CH 0.15 OEO	CH 0.06 CLEO	CH 0.1 CLEO
**Water activity (a_w_)**						
Day 2	0.890 ± 0.020 ^Aa^	0.912 ± 0.002 ^Aa^	0.918 ± 0.002 ^Ab^	0.922 ± 0.000 ^Ab^	0.922 ± 0.000 ^Ab^	0.926 ± 0.001 ^Ab^
Day 9	0.910 ± 0.004 ^Aa^	0.922 ± 0.002 ^Ba^	0.922 ± 0.002 ^Ba^	0.945 ± 0.000 ^Bb^	0.866 ± 0.010 ^Bc^	0.894 ± 0.009 ^Bc^
**TSS (°Brix)**						
Day 2	14.5 ± 0.1 ^Aa^	12.4 ± 0.2 ^Ab^	12.2 ± 0.6 ^Ab^	12.0 ± 0.2 ^Aab^	12.6 ± 0.2 ^Aab^	12.0 ± 0 ^Aab^
Day 9	14.8 ± 0.5 ^Aa^	14.0 ± 0.1 ^Ba^	15.2 ± 0.4 ^Bab^	12.7 ± 0.6 ^Abc^	17.4 ± 0.2 ^Bbc^	14.7 ± 0.3 ^Ba^
**Firmness (N)**						
Day 2	30.1 ± 4.7 ^Aa^	30.8 ± 3.7 ^Aa^	26.6 ± 2.2 ^Aa^	28.8 ± 1.6 ^Aa^	27.8 ± 1.0 ^Aa^	28.5 ± 1.4 ^Aa^
Day 9	28.5 ± 2.1 ^Aa^	22.5 ± 2.9 ^Bb^	25.3 ± 1.2 ^Aa^	24.3 ± 4.0 ^Aa^	19.4 ± 7.0 ^Ab^	24.5 ± 1.8 ^Ba^
PPO activity (U/mL) *						
Day 2	0	0	0	0	0	0
Day 9	0	0	0	0	0	0

Data expressed as mean ± standard deviation (SD) (*n* = 3). * Fresh apples had a PPO activity value of 30.1 ± 3.9 U/mL. Different lowercase letters (a–c) within the same row and different capital letters (A,B) within the same column denote significant differences (*p* < 0.05).

**Table 3:** L*, a*, b* values of untreated FC apples (C), 1.5% chitosan (CH), 1.5% chitosan + 0.06% oregano essential oil (CH 0.06 OEO), 1.5% chitosan + 0.15% oregano essential oil (CH 0.15 OEO), 1.5% chitosan + 0.06% cinnamon leaf essential oil (CH 0.06 CLEO), 1.5% chitosan + 0.1% cinnamon leaf essential oil (CH 0.1 CLEO) during storage at 4 °C.

	C	CH	CH 0.06 OEO	CH 0.15 OEO	CH 0.06 CLEO	CH 0.1 CLEO
L*						
Day 2	70.43 ± 0.66 ^Aa^	75.16 ± 1.64 ^Aa^	72.33 ± 0.705 ^Aa^	67.28 ± 3.70 ^Aab^	72.45 ± 1.49 ^Aa^	74.52 ± 1.34 ^Aac^
Day 9	71.36 ± 2.52 ^Aa^	74.60 ± 0.72 ^Aab^	71.68 ± 0.728 ^Aa^	68.62 ± 0.58 ^Aac^	72.63 ± 1.57 ^Aa^	71.36 ± 2.40 ^Aa^
a*						
Day 2	3.35 ± 1.20 ^Aa^	0.52 ± 0.09 ^Ab^	−1.04 ± 0.17 ^Ab^	−1.12 ± 0.17 ^Ab^	−0.24 ± 0.35 ^Ab^	−0.96 ± 0.27 ^Ab^
Day 9	4.22 ± 0.09 ^Aa^	0.56 ± 0.09 ^Ab^	−0.44 ± 0.99 ^Ab^	2.30 ± 1.42 ^Ba^	0.30 ± 0.80 ^Ab^	1.07 ± 1.22 ^Bb^
b*						
Day 2	24.91 ± 0.52 ^Aa^	19.10 ± 0.649 ^Aa^	19.30 ± 1.63 ^Aa^	17.96 ± 1.54 ^Ab^	18.93 ± 0.69 ^Aa^	18.40 ± 0.85 ^Aa^
Day 9	23.03 ± 0.37 ^Ba^	19.31 ± 0.541 ^Aa^	20.31 ± 2.40 ^Aa^	18.05 ± 6.28 ^Ba^	18.38 ± 0.85 ^Aa^	22.74 ± 0.64 ^Ba^

Data expressed as mean ± standard deviation (SD) (*n* = 3). Different lowercase letters (a–c) within the same row and different capital letters (A,B) within the same column denote significant differences (*p* < 0.05).

## Data Availability

The raw data supporting the conclusions of this article will be made available by the authors on request.
